# Supplementary material for: Prognostic Value of Chromatin Structure Typing in Early-Stage Non-Small Cell Lung Cancer
Source: Cancers (Basel). 2023 Jun 13;15(12):3171. doi: 10.3390/cancers15123171 (PMC10296610; doi:10.3390/cancers15123171)
Supplement: Supplementary file 1 [file cancers-15-03171-s001.zip › cancers-2407168-supplementary.pdf]

Table S1. Associations between PN and relevant clinicopathologic variables in early-stage NSCLC patients

Variables	PN low risk (%)	PN middle risk (%)	PN high risk (%)	Coefficient	*P*-value
Age, years	61.33±8.24	62.06±8.40	61.82±10.45	-0.043	0.908
Gender				0.116	0.317
Male	34 (66.7)	40 (62.5)	20 (51.3)		
Female	17 (33.3)	24 (37.5)	19 (48.7)		
Histologic type				-0.265	0.004
Adenocarcinoma	22 (43.1)	40 (62.5)	30 (76.9)		
Squamous carcinoma	29 (56.9)	24 (37.5)	9 (23.1)		
Stage				0.057	0.523
Stage I	37 (72.5)	40 (62.5)	26 (66.7)		
Stage II	14 (27.5)	24 (37.5)	13 (33.3)		
Lymph node status				0.160	0.131
Negative	46 (90.2)	51 (79.9)	29 (74.4)		
Positive	5 (9.8)	13 (20.3)	10 (25.6)		
Smoke				-0.187	0.032
No	22 (43.1)	30 (46.8)	27 (69.2)		
YES	29 (56.9)	34 (53.1)	12 (30.8)		
Recurrent or metastatic				0.216	0.007
No	43 (84.3)	41 (64.0)	23 (59)		
YES	8 (15.7)	23 (35.9)	16 (41.0)		
Adjuvant therapy				0.114	0.066
No	4 (78.4)	37 (57.8)	26 (66.7)		
YES	11 (21.6)	27 (42.2)	13 (33.3)		
Total	51 (100)	64 (100)	39 (100)		

Table S2. Univariate and multivariate analysis of combined factors as predictors of DFS in early-stage Adenocarcinoma patients

Variables	n	5-year-DFS% (95%CI)	HR (95%CI)	*P-value*	AHR (95%CI) [a]	*P-value*	*P for trend*
PN							
Diploid and CHO (PN low risk)	22	95.45 (71.87-99.35)	Ref.		Ref.		
Diploid and CHE or non-diploid and CHO (PN middle risk)	40	68.30 (49.79-81.19)	7.886 (1.022-60.873)	0.048	5.800 (0.721-46.640)	0.098	0.001
Non-diploid and CHE (PN high risk)	30	46.25 (25.93-64.36)	16.257 (2.141-123.444)	0.007	15.632 (2.005-121.903)	0.009	
PS							
Diploid and LS (PS low risk)	21	95.24 (70.72-99.32)	Ref.		Ref.		
Diploid and HS or non-diploid and LS (PS middle risk)	56	58.58 (42.87-71.34)	10.437 (1.401-77.764)	0.022	9.336 (1.211-71.982)	0.032	0.021
Non-diploid and HS (PS high risk)	15	64.00 (33.49-83.35)	9.642 (1.152-80.644)	0.037	10.336 (1.224-87.309)	0.032	
NS							
CHO and LS (NS low risk)	49	77.84 (62.56-87.48)	Ref.		Ref.		
CHO and HS or CHE and LS (NS middle risk)	38	60.48 (41.37-75.09)	1.698 (0.761-3.793)	0.196	1.891 (0.806-4.434)	0.143	0.005
CHE and HS (NS high risk)	5	40.00 (5.20-75.28)	4.419 (1.367-14.281)	0.013	8.963 (2.413-33.301)	0.001	
PNS							
0 high-risk factor (PNS low risk)	20	95.00 (69.47-99.28)	Ref.		Ref.		
1 or 2 high-risk factors (PNS middle risk)	67	62.04 (47.90-73.37)	8.680 (1.170-64.381)	0.035	6.808 (0.887-52.237)	0.065	0.001
3 high-risk factors (PNS high risk)	5	40.00 (5.20-75.28)	23.037 (2.533-209.528)	0.005	29.865 (3.231-276.057)	0.003	

PN: DNA ploidy and nucleotyping, *PS*: DNA ploidy and stroma-tumor fraction, *NS*: Nucleotyping and stroma-tumor fraction, *PNS*: DNA ploidy, nucleotyping and stroma-tumor fraction

CHO: Chromatin homogeneous, *CHE*: Chromatin heterogeneous, *LS*: Low stroma, *HS*: High stroma

[a] Cox proportional hazard model adjusted for patient age, stage, smoking and adjuvant treatment.

Table S3. Associations between PN and relevant clinicopathologic variables in early-stage Adenocarcinoma patients

Variables	PN low risk (%)	PN middle risk (%)	PN high risk (%)	Coefficient	*P*-value
Age, years	61.33±8.24	62.06±8.40	61.82±10.45	-0.043	0.908
Gender				-0.086	0.417
Male	6 (27.2)	18 (45.0)	12(40.0)		
Female	16(72.8)	22 (55.0)	18(60.0)		
Stage				0.214	0.040
Stage I	21(95.5)	29(72.5)	21(70.0)		
Stage II	1(4.5)	11(27.5)	9(30.0)		
Lymph node status				0.245	0.018
Negative	22(100)	34(85.0)	23(76.7)		
Positive	0	6(15.0)	7(23.3)		
Smoke				-0.069	0.512
No	18(81.8)	24(60.0)	26(86.7)		
YES	4(18.2)	16(40.0)	4(13.3)		
Differentiation				0.257	0.013
Well-differentiated	10(45.5)	8(20.0)	6(20.0)		
Moderately differentiated	10(45.5)	16(40.0)	12(40.0)		
Poorly differentiated	2(9.0)	16(40.0)	12(40.0)		
EGFR mutation				-0.006	0.955
No	5(22.7)	11(27.5)	8(26.7)		
YES	14(63.7)	26(65.0)	21(70.0)		
Non	3(13.6)	3(7.5)	1(3.3)		
KARS mutation				-0.031	0.793
No	16(72.8)	29(72.5)	21(70.0)		
YES	3(13.6)	2(5.0)	3(10.0)		
Non	3(13.6)	9(22.5)	6(20.0)		

Recurrent or metastatic				0.366	<0.01
No	21(95.5)	28(70.0)	15(50.0)		
YES	1(4.5)	12(30.0)	15(50.0)		
Adjuvant therapy				0.144	0.170
No	20(90.9)	23(57.5)	21(70.0)		
YES	2(9.1)	17(42.5)	9(30.0)		
Total	22(100)	40100)	30(100)		
